# Some Remarks on the Remitting Fevers of the West Indies

**Published:** 1781-10

**Authors:** 


### SECTION II.

### ESSAYS AND OBSERVATIONS.

I. *Some remarks on the Remitting fevers of the West Indies. By* Dennis Ryan, M. D. *Communicated in a letter to* Dr. Simmons, F. R. S. *and by him to the Society. Read* Sept. 17th, 1781.

IT was my intention, on my firſt arrival in this country, to keep a regular journal of every important caſe I ſhould meet with: for I was confident that ſuch a collection of facts, made without any bias or partiality for any prevailing theory or ſpeculations, would be the beſt means of giving a juſt idea of the diſeaſes of this climate; but the ſtate of health I have been in, and my having always had too great a number of patients under my care, put it out of my power to execute that plan. However, a few detached remarks and obſervations may not be altogether unacceptable. It muſt be allowed, that for ſome months paſt, this iſland has afforded an ample field for medical inquiries. In the beginning of laſt Auguſt about two thouſand eight hundred troops arrived here from England. Above four hundred of them were ſent on ſhore ſick:

ſick; and ſo great has been the mortality amongſt them for the firſt two or three months, that of all who, during that time, have been ſent to the three military hoſpitals in the neighbourhood of Kingſton, the one half has ſcarcely recovered. This may appear ſtrange to any one who is acquainted only with European hoſpitals, where in general not above one in twenty-five dies *. But the cauſes of mortality in this inſtance are ſufficiently obvious.

When the troops were firſt put on board the tranſports, in the month of March, many of them were affected with dyſentery and putrid fever. From that time till they were landed here, about five months elapſed, ſo that in this ſpace the diſeaſes with which they put to ſea, became more violent and fatal; and the ſituation of men confined ſo long on board crowded ſhips brought on them a variety of ſcorbutic complaints. Hence many of them died on the paſſage, and a great number of thoſe, who were landed at Kingſton, were exhauſted with repeated attacks of flux and fever.

Beſides this, when we arrived in Kingſton,

* The Hotel Dieu in Paris is an exception to this, for there about one out of ſix dies.

there there were no proper accommodations for the reception of the ſick; and it was impoſſible to keep them from drinking new rum. The very judicious and ſimple plan of regimental hoſpitals recommended by Robert Adair, Eſq. inſpector-general of hoſpitals, was rejected for that of a general hoſpital: hence the ſick were always too much crowded together in one place, and ſeveral loſt the opportunity of being cured whilſt they were transferred to the general hoſpital, from the place where they firſt fell ſick; but now, though late, the propriety of Mr. Adair's plan is ſeen, and it is adopted.

To the above cauſes may be juſtly added the pernicious effects of heat in this climate, which is often intolerable to people in perfect health. How far Europeans, labouring under the moſt violent diſeaſes, may ſuffer from this ſource, you may perhaps be able to form ſome idea from the few following obſervations.

The degrees of heat in an hoſpital tent at Pultney Lodge Green near Kingſton.

| Days. | | Hours. | Degrees of Fahrenheit's Thermometer. |
|---|---|---|---|
| Auguſt 22d | at | 9 A. M. | 92 |
| ——— | | 12 ——— | 96 |
| ——— | | 3 P. M. | 89 |

Auguſt

| Days. | | Hours. | Degrees of Fahrenheit's Thermometer. |
|---|---|---|---|
| Auguſt 23d | at | 8 A. M. | 89 |
| ——— | | 12 —— | 95 |
| ——— | | 1 P. M. | 93 |
| ——— | | 4 P. M. | 95 |
| —— 24th | at | 6 A. M. | 75½ |
| ——— | | 11 A. M. | 89 |
| ——— | | 1 P. M. | 93½ |
| —— 25th | at | 8 A. M. | 90 |
| ——— | | 10 A. M. | 93½ |
| ——— | | 12 —— | 94 |
| ——— | | 3½ P. M. | 93 |
| —— 26th | at | 6 A. M. | 77 |
| ——— | | 1 P. M. | 92½. |
| —— 27th | at | 1 P. M. | 95 |
| ——— | | 4 P. M. | 89 |
| —— 30th | at | 12 —— | 95 |
| —— 31ſt | at | 10 A. M. | 92 |

The thermometer was ſuſpended in the middle of the tent, and there was a free admiſſion of air on all ſides.

It is juſtly obſerved, that a man on his firſt coming to this country muſt be often at a loſs how how to proceed, particularly in the treatment of fevers; notwithſtanding he may be well acquainted with all that has been hitherto written on the diſeaſes of tropical climates.

The fevers here may be juſtly ſaid to be of a Proteiform nature, ſo great is the variety, and ſo many are the appearances which they exhibit. They frequently change the type of Quotidians and Tertians for that of the remittent or continued kind, and in a very little time prove fatal. And it ſometimes happens, that the firſt and ſecond paroxyſms are ſo ſlight, that the patient does not think it worth while to take notice of them, though the next fit is immediately attended with ſymptoms of death. I have had ſeveral caſes of this kind, where, after the ſecond or third fit, the hands and feet became quite cold, and notwithſtanding that the patients lived for a day or two after, I found it impoſſible to reſtore the heat, or prevent death, although bliſters were applied, and wine and bark, and even brandy and water given in conſiderable quantities.

Danger is ſometimes preceded by circumſtances which in general forebode no evil. A man upwards of fifty years of age, after recovering from ſome complaints for which he was first ſent to the hoſpital, told me one day that he was remarkably drowſy, and could not keep himſelf from ſleeping. Of this I took no notice. The following day he made the ſame complaint; but on his informing me, that in other reſpects he was healthy and regular, I only told him there was nothing to hinder him from indulging his propenſity to ſleep. But the next morning, to my great ſurprize, I found him ſpeechleſs, and affected with ſtartings and convulſions in his hands and arms, and in a day or two after he died.

On the contrary, in other caſes the patient ſeems to be at once ſeized with ſymptoms of the moſt dangerous tendency. Thus I was informed one morning that a ſoldier, whilſt he went about attending the ſick as orderly man, ſpoke incoherently, and ſeemed not to be right in his ſenſes; that he was well the day before, and that there was no reaſon to think that he was in liquor. On ſending for him, I obſerved that he was really delirious, and every means I tried to recover him proved ineffectual.

Moſt of the fevers which I had occaſion to treat were remittents, and they were commonly attended with bilious vomitings. The moſt violent inſtances of this kind that occurred to me, me, were three cafes, which, nearly about the fame time, were produced by intoxication. In all three the retching and vomiting were fo inceffant and obftinate, almoft from the very beginning, that nothing would remain on the ftomach; and when they fwallowed any thing, they were feized with the moft violent fpafms and convulfions. The whole body was agitated by thefe, for about the fpace of a minute, and particularly the neck and face: and death foon put an end to the fcene. I remember that two of thefe patients were fent from the cook-room, where, whilft I attended Pultney Lodge hofpital, two or three were every week feized with fevers; and the foldiers, who were obliged to go every day to Kingfton for provifions for the fick, in general fared no better: which fhould be a caution to the commanders of regiments in the Weft Indies, always to employ negroes to drefs the provifions for the fick, and undergo every other drudgery that attends an hofpital.

It is almoft unneceffary to obferve, that the method of treating fevers in Europe will by no means fuit this climate. There is in general an indication to difcharge bile from the fyftem; and hence the ufe of emetics might feem to be indicated; but in many cafes they are not only proper, but highly dangerous; for by increaſing the retching and vomiting which are already preſent, they often render it altogether impoſſible for the ſtomach to retain the medicines, which are abſolutely neceſſary to be employed. Hence it becomes an object to evacuate the bile by the inteſtines. And where the ſtomach is not too irritable, and the retching not ſevere, tartar emetic in ſmall doſes will promote a tendency this way, beſides its good effects in determining to the ſurface, and bringing on a remiſſion; but in general other laxatives are neceſſary.

Though I have had upwards of three hundred caſes under my care, ſince my arrival here, I have not ſo much as once had recourſe to the lancet. So great was the tendency to proſtration of ſtrength and putrefaction, that I conſidered not only as unneceſſary, but even dangerous. The moſt robuſt patients that I had, recovered very well without it; nor can I recollect more than one ſingle caſe, the ſymptoms of which towards the end gave me reaſon to think that ſome blood might have been taken away with advantage in the commencement of the fever. Dr. Cawley, who attended another of the military hoſpitals near Kingſton, informed me that he ſeldom or never had recourſe to venæſection, and and that moſt of the patients who were then affected with the yellow fever, were ſuch as, previous to their being ſent to the general hoſpital, had been blooded by other practitioners.

I do not however mean to inſinuate, that in the Weſt India fevers bleeding is totally to be exploded. My obſervations apply wholly to men who had been harraſſed with a variety of hardſhips during a tedious paſſage from Europe, and expoſed to ſeveral inconveniencies after their landing. Their ſituation was particular, and required a particular mode of treatment. When perſons of full, plethoric, and ſtrong habits are attacked with ſymptoms of a violent fever, bleeding is abſolutely neceſſary; but it is always to be uſed with great caution, and ſeldom after the ſecond day from the beginning of the fever.

As ſoon as the remiſſion takes place, all our hopes muſt concur in the liberal uſe of bark and wine. It is even frequently dangerous to wait for the remiſſion; and I have ſometimes thought that great advantage was derived from giving a decoction of the bark, with ſome ſpiritus mindereri, or a ſmall quantity of tartar emetic, whilſt the fever was preſent, and, when it had ceaſed, the bark in ſubſtance. Camphire is is likewiſe exceedingly proper and uſeful, both as an antiſpaſmodic and antiſeptic.

But, upon the whole, it is impoſſible to lay down any general rules which will apply in every caſe; and it requires the greateſt exertion of judgment and attention, in order to adopt ſuch meaſures as the circumſtances of each particular caſe may require.

I beg leave to ſend you the following caſes, as they may ſerve to illuſtrate ſome of the obſervations which I have made above, and afford a ſpecimen of the active practice, which, in my opinion, is often neceſſary to be adopted in this country, where the rapid progreſs of the diſeaſe calls at once for all the efforts of art to reſcue the patient from impending death.

CASE I. October 21ſt. at 4 P. M.

William Wright, aged 30, was ſeized two hours ago with coldneſs and ſhivering, which were followed by ſome heat. At preſent he is in a cold ſweat, and his pulſe is ſo low, that the pulſations can ſcarcely be perceived; his hands and feet feel cold; he complains of griping pains in his abdomen, and has had two or three ſtools, which were ſomewhat bloody, and frequently vomited bile ſince this attack began. He was ſo well this morning, that, at his own deſire, desire, he was appointed orderly-man to attend the sick; but he now says that he had a slight agueish fit last night, and another the night before, and that he concealed them, expecting that they would cease without any remedy.

Capiat omni semihora pulveris corticis Peruviani drachmam unam, cum vini uncia unica. Injiciatur statim enema ex aquâ tepida, et foveatur abdomen calida infusione florum chamæmelorum. Proinde, applicetur vesicatorium inter scapulas, et, ℞. liquoris *limonade* dicti, vini, partes æquales, utatur hac mistura pro potu communi, sed tepida fiat.

22d—He took six drachms of bark with wine yesterday evening, after which he fell asleep, and slept till about eleven at night; since that time he has taken nine drachms of bark, and drank a quart of wine and lemonade. The blister was applied, and had the desired effect. When he began to sleep last night his extremities gradually grew warm, and a general profuse sweat ensued. At present he feels no uneasiness or pain in his abdomen. This morning he had two copious stools of a greenish colour; skin cool; pulse quick and small; tongue clean; he complains of thirst; has vomited twice this morning.

Capiat

Capiat ſingulis horis pulveris corticis Peruviani drachmam unam cum vino, ut antea. Repetatur potus communis heri preſcriptus pro re nata.

23d—He has taken two ounces of bark ſince yeſterday, and had five or ſix ſtools without much pain or griping; pulſe about 80 and pretty full; thirſt leſs.

Capiat pulveris corticis Peruviani drachmam ſecunda quaque hora; et habeat indies vini libram unam.

24th—Continues to recover.

Capiat pulveris corticis Peruviani ſemunciam indies tantummodo.

Free from complaints.—Intermittantur medicamenta.

About the middle of September I had a patient, who, after labouring for three days under a continued fever, was ſeized with very nearly the ſame ſymptoms that are deſcribed in the above caſe, but he was affected with frequent bloody ſtools. However, I ordered him the bark and wine in the ſame manner; the bloody ſtools ſoon ceaſed, and he recovered, though eight and forty hours elapſed before his hands and feet began to recover their natural heat.

CASE

CASE II. October 27th.

John Perkinhorn, aged 52, complains of a great weaknefs and head-ach, with gripes and loofenefs. The day before yefterday he had a fit of the ague. He has been in the hofpital ten weeks for a variety of complaints, from which of late he has been recovering.

Capiat vefperi pulverem emeticum.

28th—Is better in every refpect.

Capiat indies pulveris corticis Peruviani femunciam et pilulam anodynam vefperi fi alvus laxa fuerit.

November 2d—He had the ague yefterday; takes his bark; ftaggers much when he fits up, and has tremulous motions in his hands and arms; fkin hot; pulfe quick.

℞ Julapii camphorati uncias fex,
Spiritus Mindereri uncias duas. M.

Capiat unciam fingulis horis. Applicetur veficatorium dorfo.

Capiat etiam pulveris corticis Peruviani drachmam unam fingulis horis, fed alternatim cum miftura fupra præfcripta.

3d—Took his medicines regularly, and his blifter rofe well; convulfive motions gone; fkin cool; pulfe regular; belly natural.

 Capiat

Capiat pulveris corticis Peruviani drachmam ſingulis horis, cum vini uncia una—intermittatur miſtura cum camphora.

4th—He has taken twelve drachms of bark ſince yeſterday morning; is again ſubject to the ſtaggering and convulſive motions, and can make no anſwer when ſpoken to; tongue dry and parched; pulſe ſtrong and quick; heat greater than natural; had two ſtools laſt night, and an involuntary one this morning.

Continuentur cortex et vinum; et capiat ſingulis horis julapii camphorati ſemunciam; capiat etiam hauſtum ſequentem veſperi.

℞ Tinct. Thebaicæ guttas triginta,

—— Japonicæ drachmas duas,

Aquæ menthæ unciam unam,

—— fontanæ uncias tres,

Syrupi ſimplicis quantum ſufficit.

Miſce ut fiat hauſtus.

5th—Has taken his medicines regularly, and ſlept well laſt night; the convulſive motions have almoſt ceaſed, and he can make diſtinct anſwers; has called for the bed-pan twice this morning; heat natural; pulſe about 80; tongue moiſt.

Continuentur julapium camphoratum et cortex; habeat indies vini libras duas.

6th—

6th—Has taken an ounce of bark ſince yeſterday, and the camphorated julap; has had about ten looſe ſtools ſince yeſterday evening; pulſe about 70; heat natural; tongue dry.

Continuentur medicamenta ut heri; ſed repetatur hauſtus anodynus ſupra præſcriptus.

7th—Has taken an ounce of bark ſince yeſterday; has had but two ſtools ſince laſt night; pulſe full, and about 70; tongue clean and moiſt.

Continuentur medicamenta ut antea.

8th—Seems to recover.—Intermittatur julapium camphoratum, continuentur cætera.

11th—Complains of nothing but weakneſs. Intermittatur cortex; habeat indies vini libram unam.

*Jamaica,*
Dec. 12th, 1781.

